# Leveraging Google Scholar to Facilitate Resident Research Reporting

**DOI:** 10.7759/cureus.19022

**Published:** 2021-10-25

**Authors:** Michael Gottlieb, Scott Heinrich, Katarzyna Gore

**Affiliations:** 1 Emergency Medicine, Rush University Medical Center, Chicago, USA

**Keywords:** google scholar, acgme, residency, scholarship, publication

## Abstract

Introduction

The Accreditation Council for Graduate Medical Education (ACGME) requires annual reporting of resident scholarly activities. However, this can be time-consuming for both residents and residency leadership and may not contain the most accurate or up-to-date information. This study sought to determine whether Google Scholar could adequately identify resident publications when compared with their ACGME self-report.

Methods

This was a cross-sectional study comparing resident Google Scholar publications with their ACGME self-reported data from 2018 to 2020. Manuscripts were only included if they were published prior to June 30, 2020, and while the participant was a resident at Rush University Medical Center. We did not count articles published prior to beginning residency. We independently collected data from the residents' self-report and Google Scholar profiles and compared the results. We assessed the overall concordance between data. When a discordant publication was identified, it was reviewed in full and discussed with the resident to ensure that it was correctly attributed to the resident. Data were presented primarily as descriptive statistics including percentages.

Results

Of 24 (96%) residents, 23 created Google Scholar profiles. There were 22 total publications. Google Scholar was concordant with self-report in 18 (78.3%) instances and discordant in five (21.7%) cases. In all five residents (n = 9 publications), the discordant publication was correctly identified by Google Scholar despite not being reported by the resident in their ACGME self-report.

Conclusion

We found that resident Google Scholar accounts resulted in the identification of publications that had not been previously reported on their ACGME self-report without missing any relevant publications.

## Introduction

The Accreditation Council for Graduate Medical Education (ACGME) stipulates that all residents participate in some form of scholarly activity prior to graduation from their residency program [1]. This provides valuable insight into the research process to develop the skills to conduct and analyze research [2-10]. Each year, residents are asked to self-report their scholarly activities for submission to the ACGME. The process of collecting the information and entering the data is generally time-consuming for residents and program faculty. There can also be difficulties with obtaining the most accurate or up-to-date information from residents.

A freely available repository of scholarly work, which can accurately track published works in real time, could be beneficial to address this challenge. Google Scholar is one such search engine that allows anyone to search for scholarly work among numerous sources, including articles, theses, books, and abstracts [11]. An individual can create a profile within Google Scholar, which would allow them to collate their publications and citations and share their research profile with others [11]. This study sought to determine whether Google Scholar could adequately identify resident publications when compared with their ACGME self-report.

This article was previously presented as a meeting abstract at the 2021 Society for Academic Emergency Medicine Scientific Assembly on May 13, 2021.

## Materials and methods

This cross-sectional study sought to compare Google Scholar with resident ACGME self-report to assess the degree of concordance with regard to publications. The study was conducted at a three-year, urban, academic emergency medicine residency program with 12 residents per postgraduate year (PGY) (36 total residents). Between August and October 2020, we asked all residents to create an account in Google Scholar using their university email. Residents were guided through the process with a brief online tutorial. After all accounts were created, we compared their publications listed in Google Scholar with those self-reported in their ACGME surveys for 2018-2020. Manuscripts were only included if they were published while they were currently a resident at Rush University Medical Center. Manuscripts published prior to residency or that had not yet been published as of June 30, 2020 (i.e., the date of the most recent ACGME survey) were excluded. Only postgraduate year (PGY) 2 and 3 residents were eligible for inclusion, as PGY 1 residents had not officially started residency until July 1, 2020. This study was deemed exempt by the Rush University Medical Center institutional review board. The authors do not have any conflicts of interest to declare.

We collected the resident name, resident PGY level, and individual PubMed identification numbers from the ACGME research tracking report for all manuscripts that met the inclusion criteria. We then independently obtained this data using their Google Scholar profile. The resulting publication data from both the self-report and Google Scholar accounts were compared. A resident’s Google Scholar profile was deemed "concordant" with their ACGME self-report if all included publications in Google Scholar were the same as those in their ACGME report. A resident’s Google Scholar profile was deemed "discordant" if one or more publications were present in only one resource (i.e., Google Scholar or the ACGME report). When a discordant publication was identified, it was reviewed in full and discussed with the resident to ensure that it was correctly attributed to the resident.

Data were presented primarily as descriptive statistics including percentages. Statistical analyses were performed using Microsoft Excel, version 16.45 (Microsoft, Inc., Redmond, WA, USA).

## Results

A total of 23 out of 24 (96%) residents created Google Scholar profiles, comprising 11 PGY 2 residents and 12 PGY 3 residents. One PGY 2 resident did not create a Google Scholar profile and was excluded from the study. That resident had no prior publications. All residents completed an ACGME self-report. Thirteen residents had a total of 22 publications (mean: 1; standard deviation: 1; range: 0-3). Six publications were reported in the 2019-2020 ACGME self-report, and seven publications were reported in the 2020-2021 ACGME self-report. Google Scholar identified an additional nine publications not listed in either ACGME self-report.

Google Scholar was concordant with ACGME self-report data in 18 out of 23 (78.3%) instances and discordant in five (21.7%) cases (Table 1). In all five residents (n = 9 publications) with discordant data, the publications were correctly identified by Google Scholar as confirmed by the residents despite not being initially reported by the resident in their ACGME self-report (Table 2). There were no instances where a publication was listed on their self-report and not identified with Google Scholar.

**Table 1:** Concordance between ACGME self-report and Google Scholar PGY, postgraduate level; ACGME, Accreditation Council for Graduate Medical Education

	PGY 2	PGY 3	Total Residents
Concordant	9	9	18
Discordant	2	3	5
Total Residents	11	12	23

**Table 2:** Publications reported by PGY level PGY, postgraduate level; ACGME, Accreditation Council for Graduate Medical Education

	PGY 2	PGY 3	Total Publications
ACGME Self-Report	3	10	13
Google Scholar	7	15	22

## Discussion

Our findings suggest that Google Scholar can adequately identify resident publications. This can have important time-saving implications for residents and residency leadership alike. Emergency medicine residency programs are required to submit an annual report to the ACGME, while many programs also are responsible for creating institution-specific annual program reports [1]. Having residents create a Google Scholar account upon entry into residency and then simply verifying the validity of the output yearly would likely make it easier for program coordinators and program directors to consolidate and record scholarly activities without sacrificing accuracy in reporting. In fact, as demonstrated in our study, it may actually lead to more accurate reporting. Moreover, this can facilitate resident updating for their curriculum vitae and provide data on their scholarly impact (i.e., total citations, h-index, i10-index). It can also be valuable for continued tracking of scholarly metrics for those planning a career in academic medicine [11-13]. Moreover, it may assist faculty and other residents with identifying their research interests to help select potential collaborators for future projects [14,15].

Importantly, using Google Scholar requires a small time commitment at the beginning of the year to set up the account and help troubleshoot any issues. However, we found that using the online tutorial substantially reduced the time involved and most residents were able to easily create their accounts. Additionally, this is only a data aggregation tool, so it is important to train residents on how to best utilize this. While all publications in Google Scholar were correctly linked to the resident, we recommend residents check their profile at least once per year to remove any publications that have been misattributed to them.

Our study has several important limitations. First, our study included a select group of emergency medicine residents at a single institution. Additional studies are needed to determine whether these findings are consistent in other institutions and among other specialties. We did not perform additional searches via PubMed or other search engines, so it is possible that additional publications may have been missed by both the ACGME self-report and Google Scholar, although we expect the risk of this is low [11]. This study also only assessed a single tool (i.e., Google Scholar). Future studies should assess other online profiles (e.g., PubMed, Microsoft Academic, ResearchGate) for aggregating resident scholarly activities. While Google Scholar is also able to identify book chapters, non-peer-reviewed articles, and other scholarship not indexed in PubMed, we did not identify any examples of these in either the residents' ACGME self-report or Google Scholar, so we are unable to comment on the accuracy for this component. Additionally, we did not track the exact time commitment to create the Google Scholar account and keep it up-to-date. Future research should assess the time commitment needed for this and compare it with the time required for the annual ACGME self-report. Finally, we did not assess the impact of Google Scholar on self-reporting. It is possible that by having a Google Scholar profile, residents may become more accurate in self-reporting for future years.

## Conclusions

In summary, we found that the creation of resident Google Scholar accounts resulted in the identification of publications that had not been previously reported on their ACGME self-report. This may help reduce the burden on resident physicians and program leadership for reporting scholarly efforts. Future research should assess this among faculty and use other online profile tools.
